# Changing Epidemiology of Influenza Infections Among Children in the Post-Pandemic Period: A Case Study in Xi’an, China

**DOI:** 10.3390/vaccines13121214

**Published:** 2025-11-30

**Authors:** Zeyao Zhao, Ning Lan, Yang Chen, Juan Yang, Jing Bai, Jifeng Liu

**Affiliations:** 1 Key Laboratory of Public Health Safety, Ministry of Education, School of Public Health, Fudan University, Shanghai 200032, China; 21111020047@m.fudan.edu.cn (Z.Z.);; 2 Department of Infectious Disease Control and Prevention, Xi’an Center for Disease Control and Prevention, Xi’an 710061, China; lanning196@foxmail.com; 3 The Second Ward of Infectious Diseases, Shangluo Central Hospital, Shangluo 726000, China; 4 Shangluo City Center for Disease Control and Prevention, Shangluo 726000, China

**Keywords:** influenza infection, pathogen co-detection, post-pandemic, paediatrics, school-based vaccination

## Abstract

Background: The epidemiology of influenza was disrupted during the COVID-19 pandemic. Following the relaxation of non-pharmaceutical interventions, influenza viruses have re-emerged and caused epidemics with shifts in age distribution and seasonality. This study aimed to characterise the post-pandemic epidemiology of influenza infections among children in Xi’an, China. Methods: A retrospective analysis of laboratory-confirmed paediatric influenza cases spanning three periods [pre-pandemic (1 January 2010–22 January 2020), intra-pandemic (23 January 2020–8 January 2023), and post-pandemic (9 January 2023–31 August 2025)] was conducted. Age-specific incidences were determined by subtypes (lineage) and compared across periods. Seasonal parameters were estimated using a generalised linear model with harmonic terms. Associations between influenza infection and risk of co-detection with other respiratory pathogens were assessed using logistic regression models. Results: Influenza peak activity in the post-pandemic period was 10-fold higher than in the intra-pandemic period. The mean age of infected children increased by 1.4 years (95% CI: 1.2–1.7), shifting towards school-aged children (6–17 years). The seasonal pattern re-established with an earlier peak (13.9 weeks earlier than the pre-pandemic period, 95% CI: 10.4–15.2) and increased amplitude (10-fold and 4-fold higher than the intra- and pre-pandemic periods, respectively). It was observed that A(H1N1)pdm09 positivity was elevated in preschool and school-aged children, whereas B/Victoria infections showed renewed susceptibility among infants [0–5 months vs. 6–35 months vs. 3–5 years vs. 6–17 years: 11.0% (95% CI: 5.1–19.8) vs. 2.8% (1.9–4.0) vs. 4.0% (3.2–5.0) vs. 5.2% (4.5–6.0); *p* = 0.00014]. Influenza infection was associated with higher risk of bacterial co-detection with *Streptococcus pneumoniae* (aOR = 1.52, 95% CI: 1.22–1.91) and *Haemophilus influenzae* (aOR = 1.46, 95% CI: 1.19–1.80), but lower risk of co-detection with SARS-CoV-2 (aOR = 0.52, 95% CI: 0.27–0.99), RSV (aOR = 0.29, 95% CI: 0.11–0.79), and parainfluenza viruses (aOR = 0.16, 95% CI: 0.04–0.65). Conclusions: The post-pandemic landscape of paediatric influenza in Xi’an has undergone substantial reconfiguration, characterised by intensified activity, altered seasonality, and a marked shift in age distribution. The increased bacterial co-detection points out the potential for more severe respiratory co-infections. These findings highlight the importance of optimising vaccination timing and prompting school-aged-children-targeted immunisation programmes in the post-pandemic era.

## 1. Introduction

The COVID-19 pandemic suppressed the seasonal influenza transmission worldwide and disrupted the long-established circulation patterns that had persisted for decades [1,2,3,4]. Widespread implementation of non-pharmaceutical interventions (NPIs) dramatically curtailed influenza transmission and resulted in a historic decline in incidence and an interruption in the natural accrual of population immunity [1,2,3,4]. With the subsequent relaxation of NPIs, influenza viruses rapidly re-emerged, offering a unique natural experiment to investigate how altered transmission dynamics reshape viral ecology and host susceptibility. This post-pandemic resurgence has been characterised by considerable heterogeneity in its seasonality and demographic impact across regions [5,6].

In China, stringent pandemic control measures maintained influenza activity at historically low levels throughout the COVID-19 period. However, the easing of border and social restrictions in late 2022 precipitated a sharp and rapid resurgence [7]. This re-establishment of viral circulation provides a critical opportunity to elucidate how prolonged suppression of transmission has reconfigured the epidemiological landscape of influenza. Such an investigation is particularly relevant for paediatric populations, who serve as major amplifiers of community transmission and as sensitive indicators of changes in epidemic dynamics [8].

Vaccination is the most effective way to prevent influenza [9]. However, the influenza vaccine is not part of China’s National Immunisation Programme [10]. Although some local governments offer complementary influenza vaccines to schoolchildren (aged 6–18 years) and older adults (aged 60 years and above), other high-priority groups, including pregnant women, infants, toddlers, and preschool children aged 3–5 years, are required to pay if choosing to be vaccinated [10]. The COVID-19 pandemic initially disrupted routine immunisation services, leading to a documented decline in coverage; subsequent public health efforts in the post-pandemic period have facilitated a recovery in vaccination activities and a rebound in coverage [11,12]. Against this backdrop of programme disruption and an evolving policy landscape, characterising the epidemiology of influenza infections among children in the post-pandemic period is essential for informing and optimising future vaccination strategies and schedules in China.

Emerging surveillance data from multiple countries have documented striking deviations from pre-pandemic norms, including atypical seasonal peaks, extended epidemic durations, and shifts in dominant influenza subtypes [13,14]. The post-pandemic period has been marked by the vigorous re-emergence of A(H1N1)pdm09 and B/Victoria lineage, often accompanied by notable changes in the age distribution of both mild and severe cases [15]. These observations suggest the presence of immunity gaps, particularly among young children who experienced critical developmental years without natural exposure to influenza viruses. Despite these reports, comprehensive, longitudinal analyses quantifying how the pandemic altered influenza epidemiology within a single, well-defined paediatric population remain scarce. Such analyses are essential to advance understanding beyond descriptive surveillance and toward a mechanistic appraisal of post-pandemic transmission dynamics.

To characterise the epidemiology of paediatric influenza infections in the post-pandemic period, we conducted a retrospective analysis leveraging the long-term sentinel surveillance data from Xi’an, China, spanning 2010–2025. Specifically, we aimed to describe temporal trends in influenza activity and determine the changes in seasonality, to quantify the subtype-specific infection risks for different-aged children, and to assess the risks of co-detection with other respiratory pathogens among influenza-infected children.

## 2. Materials and Methods

### 2.1. Study Design and Population

This study employs a retrospective cohort design, utilising routinely collected surveillance data to assess the impact of the COVID-19 pandemic and its aftermath on the epidemiology of paediatric influenza infections in Xi’an, China. Laboratory-confirmed influenza cases were retrospectively analysed across three distinct periods: the pre-pandemic period, the intra-pandemic period, and the post-pandemic period. This design facilitates comparisons of influenza incidence, seasonal patterns, age distribution, and co-detection of respiratory pathogens across the different phases of the pandemic. The study population includes children under 18 years presenting with influenza-like illness. Data were sourced from the national sentinel hospital-based influenza surveillance network in Xi’an, China, which monitors influenza-like illness (ILI) and conducts laboratory confirmation of influenza infections [13].

### 2.2. Study Setting

This study was conducted in Xi’an, the capital of Shaanxi Province in northwestern China, with a population of approximately 13 million [16]. The city experiences a temperate climate with distinct seasons, creating conditions conducive to seasonal influenza epidemics that typically peak in winter [13]. Data were obtained from the national sentinel hospital-based influenza surveillance network [13,17], which comprises multiple hospitals reporting weekly on ILI cases and providing laboratory-confirmed influenza results. Surveillance was performed across nine sentinel hospitals representing diverse districts of Xi’an: Xi’an Central Hospital, Xi’an People’s Hospital (Xi’an Fourth Hospital), Air Force 986 Hospital, Xi’an First Hospital, Xi’an Children’s Hospital, Xi’an Twelfth Hospital, Xi’an Yanliang District People’s Hospital, Huyi District People’s Hospital, and Xi’an Eighth Hospital. These sentinel sites include general hospitals, a specialised children’s hospital, and facilities from both urban and suburban districts, thereby ensuring comprehensive demographic and geographic coverage.

### 2.3. Data Sources

Weekly reports were obtained from the national sentinel hospital-based influenza surveillance network, which provided data on the number of laboratory-confirmed influenza cases by virus subtype (lineage) and the number of specimens tested [13]. Each week, sentinel hospitals in Xi’an reported to a centralised online system the number of patients presenting with influenza-like illness (ILI) and total outpatient or emergency department visits. Patients with ILI were identified according to a standard case definition: fever ≥ 38 °C with cough or sore throat in the absence of another diagnosis, with the onset of symptoms occurring within the current acute respiratory illness [18]. Virus isolation was performed using Madin-Darby canine kidney (MDCK) cells and specific pathogen-free (SPF) chicken embryos at the Xi’an Centers for Disease Control and Prevention (CDC) [18]. Influenza virus typing and subtyping were conducted using conventional/real-time reverse transcription PCR (RT-PCR) following standardised protocols at the Xi’an CDC [13,18]. Since 2024, Xi’an has implemented multipathogen surveillance for respiratory samples collected from ILI patients. The testing panel included ten respiratory viruses, including SARS-CoV-2, influenza viruses, respiratory syncytial virus (RSV), adenovirus, human metapneumovirus, parainfluenza virus, seasonal coronaviruses, bocavirus, rhinovirus, and enterovirus, as well as *Mycoplasma pneumoniae*, etc.

### 2.4. Study Variables, Measures, and Outcomes

The key study variables include influenza infection status (positive or negative), determined through RT-PCR; age group (e.g., infants, preschool-aged children, and school-aged children); influenza virus subtype (e.g., A(H1N1)pdm09, B/Victoria, etc.); co-detection of other respiratory pathogens, including SARS-CoV-2, RSV, *Streptococcus pneumoniae*, and *Haemophilus influenzae*; and seasonality parameters, such as peak timing, epidemic amplitude, and periodicity. The primary measures in this study are influenza infection rates, calculated as the percentage of positive influenza tests by age group and virus subtype for each study period; co-detection rates with other respiratory pathogens, assessed using logistic regression models; seasonal parameters, including the timing of peak influenza activity, epidemic amplitude, and periodicity, estimated using a generalised linear model (GLM); and bacterial co-detection odds ratios, reflecting the likelihood of co-detection with bacteria like *Streptococcus pneumoniae* and *Haemophilus influenzae* during influenza infection. The primary outcomes include age-specific influenza infection rates, stratified by virus subtype and study period (pre-, intra-, and post-pandemic); changes in seasonal influenza patterns, such as variations in epidemic amplitude and timing of peak influenza activity across the three periods; risk of bacterial co-detection, including pathogens like *Streptococcus pneumoniae* and *Haemophilus influenzae*, during influenza infection for the post-pandemic period; and shifts in the age distribution of influenza cases, with particular attention to the increase in influenza cases among school-aged children in the post-pandemic period.

### 2.5. Definition of Study Periods

The study period was divided into three distinct phases reflecting the implementation and relaxation of COVID-19 control measures in Xi’an:

Pre-pandemic period (1 January 2010–22 January 2020): Baseline influenza activity before the pandemic of SARS-CoV-2.

Intra-pandemic period (23 January 2020–8 January 2023): Period during which public health and social measures were implemented by the government (including lockdowns and social distancing, etc., http://xawjw.xa.gov.cn/, accessed on 10 November 2023).

Post-pandemic period (9 January 2023–31 August 2025): Period following major policy relaxation, capturing the resurgence of influenza transmission (https://www.nhc.gov.cn, accessed on 10 November 2023).

### 2.6. Statistical Analysis

Demographic and clinical characteristics of the study population were summarised and compared across the three epidemic periods (pre-, intra-, and post-). Continuous variables were compared using the Kruskal–Wallis test. Categorical variables were compared using the Chi-square test or Fisher’s exact test as appropriate. A two-sided *p*-value of <0.05 was considered statistically significant.

Age-specific positive rates were calculated as (number of positive tests/total number of tests) × 100% for each age group, with 95% confidence intervals (CI) derived from the exact binomial method. The absolute change in positive rates was calculated as the difference in rates between the post-pandemic and reference periods (pre- or intra-pandemic). Statistical significance of these changes was assessed using Chi-square test or Fisher’s exact test, as appropriate. The age distribution of cases was compared across periods using cumulative distribution functions.

Seasonality was quantified using a generalised linear model (GLM) with harmonic terms to estimate epidemic amplitude, timing, and periodicity [13,19]. The fitted model was:

(1) $$flu_{i}\left(t\right)=a_{i}+b_{i}\times \cos\left(2\pi t/52.17\right)+c_{i}\times \sin\left(2\pi t/52.17\right)+d_{i}\times \cos\left(4\pi t/52.17\right)+e_{i}\times \sin\left(4\pi t/52.17\right)+\varepsilon_{i}\left(t\right)$$

where *flu_i_*(*t*) represents annual standardised influenza activity for period *i*; *t* is epidemiological week; *a_i_*, *b_i_*, *c_i_*, *d_i_* and *e_i_* are fitted seasonality coefficients; and *ε_i_*(*t*) are errors. Amplitude, peak timing, and semi-annual periodicity were derived from the model coefficients *a_i_* − *e_i_*. Confidence intervals on estimates of relative amplitude, peak timing, and periodicity ratio were obtained by fitting seasonal regression models to 1000 datasets resampled from the original data by bootstrap, which accounts for autocorrelation in weekly influenza incidences [13,19].

Generalised linear models were used to calculate unadjusted and adjusted odds ratios (ORs) with 95% confidence intervals (CIs) for the association between influenza infection (independent variable) and the risk of co-detection of other respiratory pathogens (dependent variables). The initial model assessed crude associations, followed by a multivariable model adjusted for age and sex to control for potential confounding.

## 3. Results

### 3.1. Characteristics of the Study Population and Influenza Cases Across Periods

#### 3.1.1. Characteristics of Study Population

A total of 39,594 paediatric ILI cases with laboratory diagnostic results were included, comprising 26,727 pre-pandemic, 6243 intra-pandemic, and 6624 post-pandemic cases.

The mean age of ILI cases increased significantly from 4.69 years (SD 3.75) pre-pandemic to 6.47 years (SD 4.19) post-pandemic (mean difference: 1.79 years, 95% CI: 1.68–1.90, *p* < 0.001). A marked decline in cases among children aged 6–35 months (31.9% vs. 16.5%, *p* < 0.001) was observed, accompanied by a relative increase among school-aged children (32.2% vs. 52.7%, *p* < 0.001; Table A1).

#### 3.1.2. Characteristics of Laboratory-Confirmed Influenza Cases

**Figure A1:**
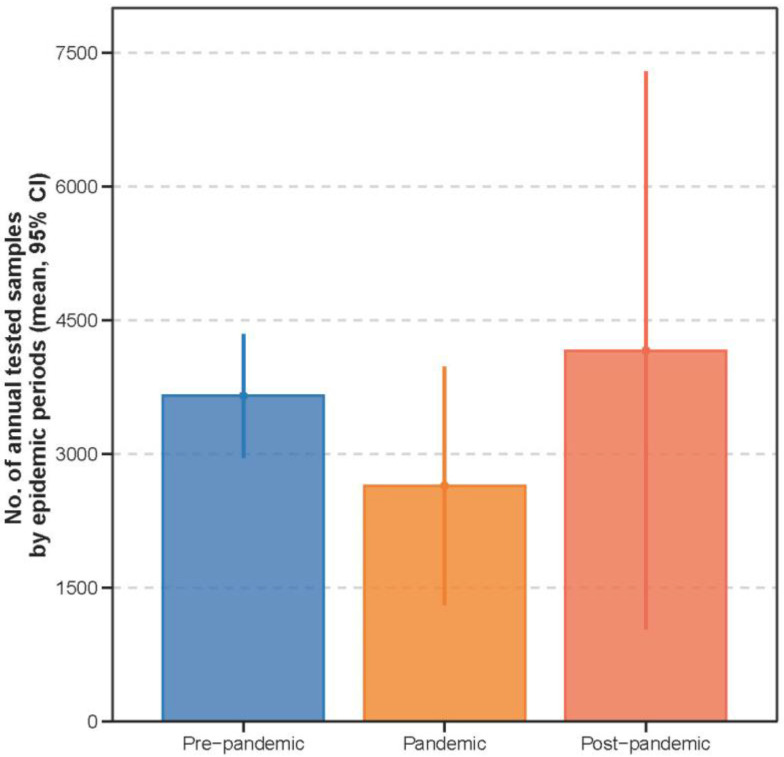
Comparison of annual tested samples across periods.

Among 6216 laboratory-confirmed influenza cases, 4508 occurred pre-pandemic, 460 during the pandemic, and 1248 post-pandemic (Table 1), with a similar number of samples tested annually across the three periods (median: 2945 samples per year; Figure A1). Significant differences were observed across periods in age, age group distribution, seasonality, and influenza subtype (*p* < 0.001 for all comparisons). The mean age of influenza-positive children increased from 5.53 years pre-pandemic to 6.66 years intra-pandemic and 7.00 years post-pandemic. No significant variation in the sex distribution was detected across periods (*p* = 0.704).

#### 3.1.3. Shifts in Dominant Influenza Subtypes

**Figure 1:**
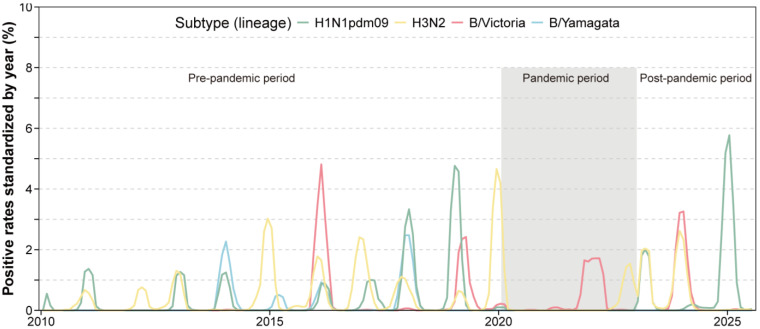
Time series of influenza positive rates across the pre-, intra- and post-pandemic periods.

Throughout the pandemic, influenza B/Victoria was predominant, accounting for 64.6% of influenza-infected cases, while A(H1N1)pdm09 was nearly absent and B/Yamagata completely undetected. In the post-pandemic period, A(H1N1)pdm09 re-emerged as the dominant subtype (48.2%), followed by A(H3N2) (27.6%) and B/Victoria (24.1%); B/Yamagata remained undetected (Table 1). Further analyses revealed successive subtype-driven epidemic waves after 2023 (Figure 1): a spring 2023 wave dominated by A(H3N2) and A(H1N1)pdm09, a B/Victoria wave in winter 2023–2024, and another major A(H1N1)pdm09 wave in late 2024 to early 2025.

### 3.2. Re-Emergence of Seasonal Patterns Post-Pandemic

Seasonal influenza activity was substantially disrupted during the pandemic but demonstrated progressive re-establishment thereafter (Table 1 and Table 2). During the pandemic, winter peaks diminished (32.1% vs. 43.1% pre-pandemic), while autumn activity increased (37.0% vs. 29.0%). In the post-pandemic period, winter epidemics (37.8%) and elevated spring activity (25.2% vs. 19.5%) were observed. The annual epidemic amplitude was significantly greater for the post-pandemic (0.012585, 95% CI: 0.009701–0.015822) period than both the pre-pandemic (0.002871, 95% CI: 0.002177–0.003710) and intra-pandemic (0.001232, 95% CI: 0.000792–0.001818) periods. While the seasonal peak occurred earlier by 13.9 weeks (95% CI: 10.4–15.2) compared with the pre-pandemic period, the epidemic duration returned to a range similar to that observed before the COVID-19 pandemic (Table 2, Table A4).

### 3.3. Shifts in the Age Distribution of Influenza Infections

#### 3.3.1. Age Profiles of Infection Across Periods

**Figure 2:**
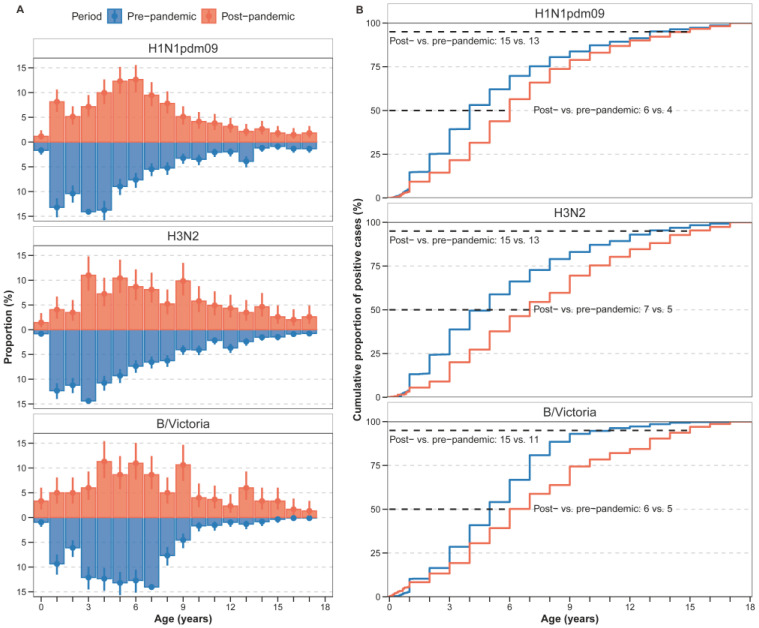
Age distribution of paediatric influenza cases in the pre- and post-pandemic period. (**A**) Proportion of age-specific cases among total influenza cases, presented as percentages with 95% confidence intervals (95% CI). (**B**) Cumulative distribution of cases by age group for each time period. The lines represent the cumulative frequency of cases across age groups for the pre-pandemic (blue) and post-pandemic (red) periods.

**Figure A2:**
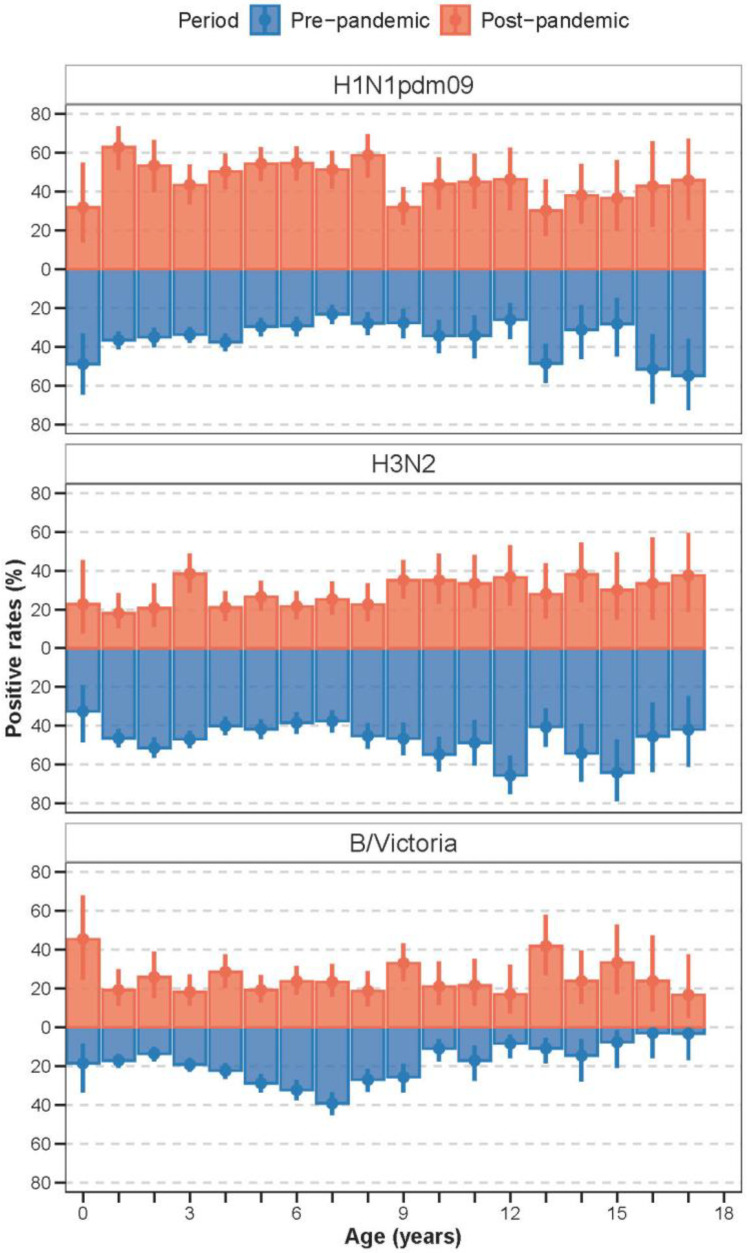
Age-specific seropositive rates for different subtypes (lineages) in the pre- and post-pandemic periods. The positive rates were presented as percentages with 95% confidence intervals (95% CI).

In the pre-pandemic period, younger children bore the greatest burden of influenza A subtypes compared with influenza B (Figure 2A and Figure A2). Specifically, for B/Victoria, infection rates peaked among 5–7-year-olds before the pandemic (Figure A2). In the post-pandemic period, positive rates peaked among young infants aged below 1 year for B/Victoria. In addition, the post-pandemic period exhibited a pronounced age shift toward school-aged children, especially for A(H1N1)pdm09 and B/Victoria (Figure 2A and Figure A2). For A(H1N1)pdm09, the positive rates among 3–6-year-olds (kindergarten children) significantly increased. Similar trends were observed for B/Victoria, with increased proportions in kindergarten children and school-aged children (Figure 2A and Figure A2).

**Figure A3:**
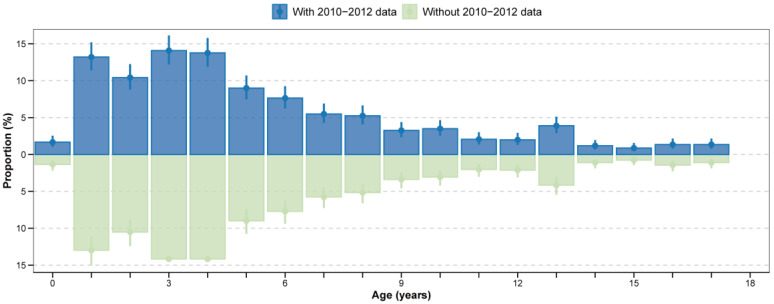
Age distribution of paediatric A(H1N1)pdm09 cases in the pre-pandemic period with and without 2010−2012 data. Proportion of age-specific cases among total influenza cases, presented as percentages with 95% confidence intervals (95% CI).

To quantify this shift, we compared the median age and the 95th percentile of the age distribution between periods (Figure 2B, Figure A3). The median age of infection increased for all (sub)types. For H1N1pdm09, the median age increased from 4 years pre-pandemic to 6 years post-pandemic. For H3N2, the median increased from 5 years to 7 years, and for B/Victoria, from 5 years to 6 years. Moreover, the 95th percentile of the age distribution expanded, with the upper age limit rising from 13 to 15 years pre-pandemic to 15 years across all subtypes post-pandemic.

#### 3.3.2. Changes in Age-Specific Positive Rates

**Figure 3:**
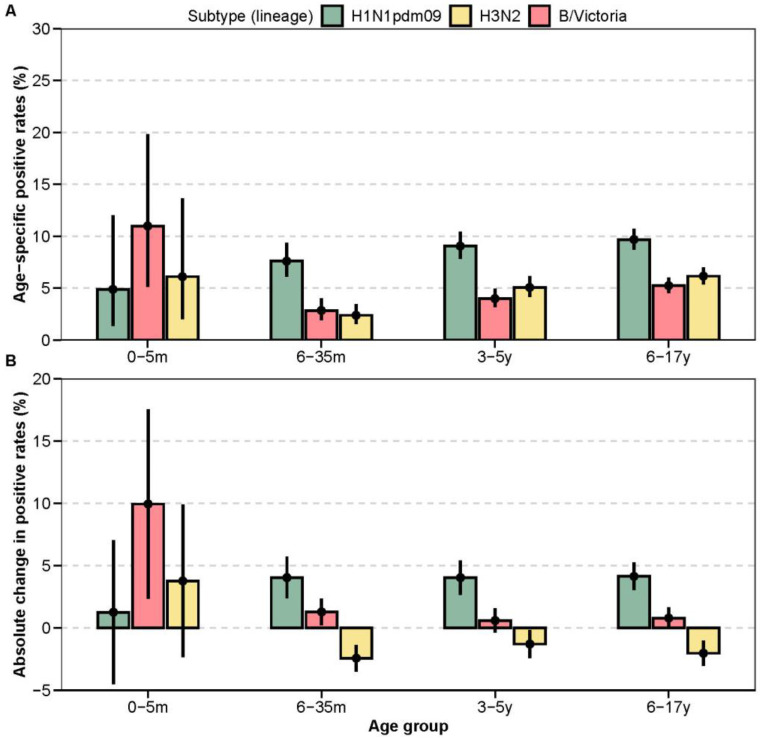
Age-specific changes in influenza positive rates. (**A**) Subtype-specific positive rates by age group in the post-pandemic period. Point estimates and corresponding 95% confidence intervals (95% CI) are shown. (**B**) Absolute change in subtype-specific positive rates in the post-pandemic period compared to the pre-pandemic baseline.

In the post-pandemic period, age-specific influenza positive rates exhibited distinct subtype-specific patterns relative to pre-pandemic levels (Figure 3A,B; Table A3 and Table A5). For A(H1N1)pdm09, positive rates increased significantly across all age groups: +4.0% (6–35 months, *p* < 0.001), +4.0% (3–5 years, *p* < 0.001), and +4.1% (6–17 years, *p* < 0.001). Age-specific positive rates rose progressively from infancy to adolescence (6–35 months: 7.6%, 95% CI: 6.1–9.3; 3–5 years: 9.0%, 95% CI: 7.8–10.4; 6–17 years: 9.7%, 95% CI: 8.7–10.7).

In contrast, the post-pandemic epidemiology of H3N2 was characterised by significantly lower positive rates in older children. A significant decrease was observed in the 6–35-month (−2.4%, *p* < 0.001), 3–5-year (−1.3%, *p* = 0.034), and 6–17-year (−2.0%, *p* < 0.001) age groups compared to the pre-pandemic era. While among infants aged 0-5 months, the H3N2 positive rate increased slightly, though not significantly (+3.8%, *p* = 0.144). For influenza B/Victoria, the most pronounced change was a significant increase in positivity among infants aged 0–5 months (+9.9%, *p* < 0.001). More modest but significant increases were also observed in the 6–35-month age group (+1.3%, *p* = 0.003).

### 3.4. Co-Detection of Other Respiratory Pathogens Among Influenza-Positive Cases

**Figure 4:**
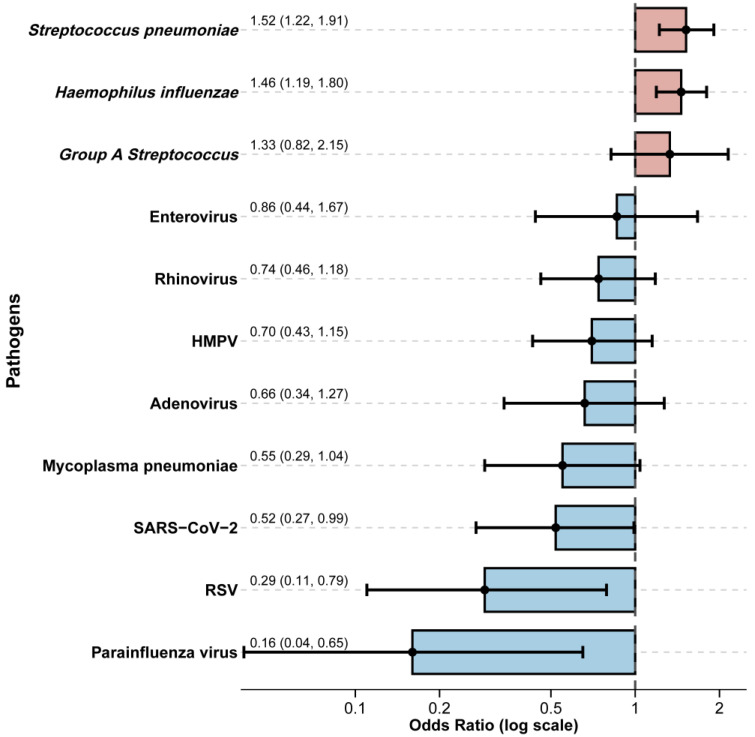
Association between influenza virus infection and co-detection of other respiratory pathogens. Odds ratios (ORs) and corresponding 95% confidence intervals (95% CI) are presented, derived from generalised models adjusting for age and sex. Bars show the point estimates of adjusted odds ratios for co-detection groups: non-bacterial pathogens (viral or mycoplasma; blue) and bacterial pathogens (red).

We compared the risk of codetection of other respiratory pathogens among influenza cases. Significant differences in risk of codetection of other pathogens between the influenza-infected (*n* = 229) and non-influenza (*n* = 1119) groups were observed (Table A2). Notably, detection rates for respiratory syncytial virus (RSV) (0.9% vs. 4.6%, *p* = 0.014) and parainfluenza virus (0.4% vs. 5.4%, *p* = 0.002) were significantly lower in influenza-infected children. Conversely, the bacterial pathogens *Streptococcus pneumoniae* (30.1% vs. 19.0%, *p* < 0.001) and *Haemophilus influenzae* (40.2% vs. 28.6%, *p* = 0.001) were detected more frequently in the influenza-infected group. To account for potential confounders, we performed a multivariable logistic regression analysis adjusted for age and sex (Figure 4). This analysis confirmed significant negative associations between influenza infection and co-detection with SARS-CoV-2 (adjusted Odds Ratio [aOR] = 0.52, 95% CI: 0.27–0.99) and parainfluenza virus (aOR = 0.16, 95% CI: 0.04–0.65). A trend towards a negative association was observed with RSV (aOR = 0.29, 95% CI: 0.11–0.79). In contrast, significant positive associations were identified with bacterial co-detection. Influenza infection was independently associated with increased odds of co-detection with *Streptococcus pneumoniae* (aOR = 1.52, 95% CI: 1.22–1.91) and *Haemophilus influenzae* (aOR = 1.46, 95% CI: 1.19–1.80). No significant associations were observed for other tested viruses, including adenovirus, human metapneumovirus, rhinovirus, and enterovirus, or for *Mycoplasma pneumoniae* and *Group A Streptococcus*.

## 4. Discussion

This long-term sentinel surveillance study reveals that the epidemiology of paediatric influenza infections was profoundly reshaped following the COVID-19 pandemic. We observed intensified influenza activity with an earlier epidemic peak and a marked demographic shift toward school-aged children. Subtype-specific risks also diverged, with A(H1N1)pdm09 disproportionately affecting older children and a resurgence of susceptibility to B/Victoria among young infants aged 0–5 months. Furthermore, influenza infection was associated with an increased risk of bacterial co-detection, such as S. pneumoniae and H. influenzae, but a decreased risk of co-detection with other key respiratory viruses, including RSV and SARS-CoV-2.

The robust resurgence of influenza following the relaxation of COVID-19 control measures mirrors global trends of rapid viral reintroduction into populations with reduced immunity [7,15]. The alternating predominance of A(H1N1)pdm09 and A(H3N2) observed in Xi’an aligns with surveillance data from other regions of China and East Asia [5]. The continued absence of B/Yamagata is consistent with global findings suggesting possible lineage extinction during the pandemic [20,21]. The elevated intensity of post-pandemic epidemics likely reflects the accumulation of susceptible individuals across several paediatric birth cohorts, resulting from prolonged interruption of viral circulation and waning immunity [15,22]. Rebuilding and maintaining population immunity will require sustained vaccination coverage, particularly in younger cohorts who missed natural exposures during the pandemic, to prevent outbreaks among the high-risk susceptible population in the coming years.

Our findings also demonstrate that influenza seasonality not only re-emerged but also became more pronounced, with epidemics peaking earlier and lasting longer than in the pre-pandemic era. Comparable intensification has been documented in Japan and Europe, where earlier peaks have been attributed to increased post-pandemic mobility and faster seeding of epidemics [7]. The earlier onset observed in Xi’an may similarly reflect post-pandemic synchronisation of transmission dynamics between northern and southern China, enhancing interregional viral spread. The prolonged epidemic duration likely reflects broader immunity gaps across age groups, sustaining community transmission over an extended period [23,24,25]. Earlier and prolonged epidemic activity calls for advancing the timing of annual influenza vaccination campaigns and reinforcing early-warning surveillance systems to anticipate and mitigate earlier peaks.

A key observation is the substantial upward shift in the age distribution of cases toward school-aged children. This shift underscores the profound cohort-specific effects of transmission interruption. The near-absence of influenza between 2020 and 2022 likely delayed primary infections and created a large reservoir of immunologically naïve older children [23,25]. Comparable shifts toward older age groups have been documented in Australia, the United States, and Europe [23,24,25,26]. These findings highlight the importance of maintaining high influenza vaccination coverage in school-aged children, who may now act as primary amplifiers of community transmission in the post-pandemic setting [27]. Expanding school-based influenza vaccination programmes and integrating influenza education into school health policies could substantially reduce transmission and protect both paediatric and adult populations [28,29].

The distinct age and subtype patterns observed, particularly the resurgence of A(H1N1)pdm09 among preschool and school-aged children and the renewed vulnerability of infants to B/Victoria, suggest heterogeneous immunity gaps across birth cohorts and that the “immunological debt” incurred during the pandemic is not uniform [30]. Infants born during this period may have received lower levels of transplacentally transferred maternal antibodies due to minimal community circulation of influenza viruses, increasing their vulnerability [31]. Conversely, older children, who missed one or more natural exposures, might now face increased infection risk from A(H1N1)pdm09. These findings underscore the need for integrated seroepidemiological studies to map population-level susceptibility and inform vaccination strategies. Tailored vaccination strategies are needed, emphasising maternal immunisation during pregnancy and early childhood vaccination to bridge cohort-specific immunity gaps and protect the most vulnerable infants.

The pathogen co-detection patterns provide further evidence of a rebalanced respiratory ecosystem. The negative associations between influenza and other respiratory viruses, such as RSV and parainfluenza, are consistent with viral interference mechanisms mediated by interferon-induced innate immune responses [32,33,34]. The elevated detection of *Streptococcus pneumoniae* and *Haemophilus influenzae* among influenza cases, however, reflects enhanced bacterial colonisation or co-infection potential, consistent with previous studies showing influenza-associated bacterial superinfection as a major cause of paediatric morbidity [35,36,37]. These findings emphasise the importance of integrated surveillance and vaccination strategies that address both viral and bacterial respiratory pathogens. Integrated viral-bacterial surveillance and promotion of pneumococcal and *H. influenzae type b* vaccination can reduce influenza-related bacterial complications and paediatric morbidity in the post-pandemic era.

While our study provides a comprehensive analysis of the post-pandemic influenza landscape, several limitations must be considered when interpreting the findings. To fully understand the long-term dynamics, continued surveillance over multiple seasonal cycles is essential to determine whether these patterns represent a permanent reconfiguration or a transient post-pandemic shift. Expanding studies to diverse geographical and sociodemographic settings will help build a comprehensive national picture of influenza epidemiology. The introduction of multipathogen surveillance in 2024 enables more detailed investigation of viral and bacterial co-infections. However, these data are only available from 2024 onwards due to the implementation of multipathogen surveillance in Xi’an, limiting the ability to make comprehensive comparisons across the entire study period. This limitation restricts inferences about temporal trends in co-infections and their potential impact on influenza transmission dynamics, and future studies extending these analyses over longer periods could clarify temporal trends and their impact on influenza transmission. Additionally, integrating data on disease severity and clinical outcomes, such as hospitalisations, complications, and mortality, will be critical to assess the full burden of influenza and associated co-infections. Finally, prospective studies incorporating detailed virological, microbiological, and immunological measurements are warranted to elucidate the biological mechanisms underlying observed co-detection patterns and to inform targeted prevention and intervention strategies.

## 5. Conclusions

In summary, paediatric influenza infections have rebounded strongly following the COVID-19 pandemic, with intensified activity and earlier epidemic peaks, and shifted age distribution. These changes underscore the dynamic nature of post-pandemic respiratory pathogen ecology and the critical need for integrated, adaptive surveillance systems. Sustained monitoring of influenza transmission, susceptibility, and interaction with other pathogens will be essential to guide vaccination strategies and mitigate the resurgence of influenza-associated morbidity in the paediatric population.

## Figures and Tables

**Table 1:** Demographic characteristics of influenza cases across pre-pandemic, intra-pandemic, and post-pandemic periods.

Characteristics	Pre-Pandemic (N = 4508)	Pandemic (N = 460)	Post-Pandemic (N = 1248)	*p*-Value
Age (years, mean, sd)	5.53 (3.69)	6.66 (3.20)	7.00 (4.12)	<0.001
Age group (*n*, %)				<0.001
0–5 m	29 (0.6)	1 (0.2)	18 (1.4)	
6–35 m	946 (21.0)	41 (8.9)	140 (11.2)	
3–5 y	1606 (35.6)	123 (26.7)	354 (28.4)	
6–17 y	1927 (42.7)	295 (64.1)	736 (59.0)	
Sex (*n*, %)				0.704
Female	2036 (45.2)	215 (46.7)	555 (44.5)	
Male	2472 (54.8)	245 (53.3)	693 (55.5)	
Season (*n*, %)				<0.001
Spring	846 (18.8)	101 (22.0)	348 (27.9)	
Summer	46 (1.0)	12 (2.6)	15 (1.2)	
Autumn	413 (9.2)	166 (36.1)	105 (8.4)	
Winter	3203 (71.1)	181 (39.3)	780 (62.5)	
Subtype (*n*, %)				<0.001
H1N1pdm09	1257 (27.9)	3 (0.7)	602 (48.2)	
H3N2	1710 (37.9)	160 (34.8)	345 (27.6)	
B/Victoria	834 (18.5)	297 (64.6)	301 (24.1)	
B/Yamagata	707 (15.7)	0 (0.0)	0 (0.0)	

**Table 2:** Comparison of influenza seasonality metrics in children across the pre-pandemic, pandemic, and post-pandemic periods.

Parameters	Pre-Pandemic	Pandemic	Post-Pandemic
Amplitude (95% CI)	0.002871 (0.002177–0.003710)	0.001232 (0.000792–0.001818)	0.012585 (0.009701–0.015822)
Semi-annual amplitude (95% CI)	0.002041 (0.001300–0.002855)	0.000480 (0.000223–0.001050)	0.008407 (0.005799–0.011423)
Annual peak time (weeks, 95% CI)	19.6 (17.0–21.8)	2.7 (0.2–5.8)	5.7 (4.9–6.6)
Semi-annual periodicity (%, 95% CI)	41.5 (30.0–52.3)	28.0 (13.6–48.7)	40.0 (36.2–43.6)
Epidemic duration (weeks, 95% CI)	17.0 (11.5–21.0)	9.0 (7.5–13.0)	18.0 (12.0–19.0)

## Data Availability

The original contributions presented in this study are included in the article. Further inquiries can be directed to the corresponding authors.

## Abbreviations

The following abbreviations are used in this manuscript:

NPIs	Non-pharmaceutical interventions
COVID-19	Corona Virus Disease 2019
RSV	Respiratory syncytial virus

## Appendix A

**Table A1:** Demographic and clinical characteristics of the study population across pre-, during-, and post-pandemic periods.

Characteristics	Pre-Pandemic (N = 26,727)	Pandemic (N = 6243)	Post-Pandemic (N = 6624)	*p*-Value
Age (years, mean, sd)	4.69 (3.75)	4.87 (3.43)	6.47 (4.19)	<0.001
Age group (*n*, %)				<0.001
0–5 m	386 (1.4)	68 (1.1)	82 (1.2)	
6–35 m	8520 (31.9)	1616 (25.9)	1092 (16.5)	
3–5 y	9227 (34.5)	2320 (37.2)	1956 (29.5)	
6–17 y	8594 (32.2)	2239 (35.9)	3494 (52.7)	
Sex (%)				0.08
Female	11,706 (43.8)	2775 (44.4)	3000 (45.3)	
Male	15,021 (56.2)	3468 (55.6)	3624 (54.7)	

Note that among 6216 laboratory-confirmed influenza cases, 4508 occurred pre-pandemic, 460 during the pandemic, and 1248 post-pandemic, with similar annual tested samples across the three periods.

**Table A2:** Comparison of pathogen co-detection risks between influenza-infected and non-infected groups.

Characteristics	Influenza Non-Infected (N = 1119)	Influenza Infected (N = 229)	*p*-Value
Viruses			
SARS-CoV-2 (*n*, %)			0.061
No	1059 (94.6)	224 (97.8)	
Yes	60 (5.4)	5 (2.2)	
Respiratory syncytial virus (*n*, %)			0.014
No	1067 (95.4)	227 (99.1)	
Yes	52 (4.6)	2 (0.9)	
Adenovirus (*n*, %)			0.326
No	1077 (96.2)	224 (97.8)	
Yes	42 (3.8)	5 (2.2)	
Human metapneumovirus (*n*, %)			0.209
No	1048 (93.7)	220 (96.1)	
Yes	71 (6.3)	9 (3.9)	
Parainfluenza virus (*n*, %)			0.002
No	1059 (94.6)	228 (99.6)	
Yes	60 (5.4)	1 (0.4)	
Common coronavirus (*n*, %)			0.677
No	1114 (99.6)	229 (100.0)	
Yes	5 (0.4)	0 (0.0)	
Bocavirus (*n*, %)			1.00
No	1113 (99.5)	228 (99.6)	
Yes	6 (0.5)	1 (0.4)	
Rhinovirus (*n*, %)			0.258
No	1045 (93.4)	219 (95.6)	
Yes	74 (6.6)	10 (4.4)	
Enterovirus (*n*, %)			0.839
No	1089 (97.3)	224 (97.8)	
Yes	30 (2.7)	5 (2.2)	
Mycoplasma and bacteria			
*Mycoplasma pneumoniae* (*n*, %)			0.09
No	1063 (95.0)	224 (97.8)	
Yes	56 (5.0)	5 (2.2)	
Group A streptococcus (*n*, %)			0.359
No	1082 (96.7)	218 (95.2)	
Yes	37 (3.3)	11 (4.8)	
*Bordetella pertussis* (*n*, %)			0.763
No	1118 (99.9)	228 (99.6)	
Yes	1 (0.1)	1 (0.4)	
*Streptococcus pneumoniae* (*n*, %)			<0.001
No	906 (81.0)	160 (69.9)	
Yes	213 (19.0)	69 (30.1)	
*Haemophilus influenzae* (*n*, %)			0.001
No	799 (71.4)	137 (59.8)	
Yes	320 (28.6)	92 (40.2)	

**Table A3:** Age-specific positive rates for the post-pandemic period and absolute change in positive rates compared with the pre-pandemic period.

Age Group	Subtype	Post-Pandemic Positive Rates (95% CI)	Absolute Change in Positive Rates (95% CI)	*p*-Value
0–5 m	B/Victoria	11.0 (5.1, 19.8)	9.9 (2.4, 17.5)	<0.001
0–5 m	H1N1pdm09	4.9 (1.3, 12.0)	1.3 (−4.5, 7.0)	0.827
0–5 m	H3N2	6.1 (2.0, 13.7)	3.8 (−2.4, 9.9)	0.144
6–35 m	B/Victoria	2.8 (1.9, 4.0)	1.3 (0.2, 2.3)	0.003
6–35 m	H1N1pdm09	7.6 (6.1, 9.3)	4.0 (2.4, 5.7)	<0.001
6–35 m	H3N2	2.4 (1.6, 3.5)	−2.4 (−3.5, −1.4)	<0.001
3–5 y	B/Victoria	4.0 (3.2, 5.0)	0.6 (−0.4, 1.6)	0.226
3–5 y	H1N1pdm09	9.0 (7.8, 10.4)	4.0 (2.7, 5.4)	<0.001
3–5 y	H3N2	5.1 (4.1, 6.1)	−1.3 (−2.4, −0.2)	0.034
6–17 y	B/Victoria	5.2 (4.5, 6.0)	0.8 (−0.1, 1.7)	0.073
6–17 y	H1N1pdm09	9.7 (8.7, 10.7)	4.1 (3.0, 5.2)	<0.001
6–17 y	H3N2	6.2 (5.4, 7.0)	−2.0 (−3.0, −1.0)	<0.001

**Table A4:** Comparison of influenza seasonality metrics in the pre-pandemic period with and without 2010–2012 data.

Parameters	With 2010–2012 Data	Without 2010–2012 Data
Amplitude (95% CI)	0.002871 (0.002177–0.003710)	0.001868 (0.000564–0.003588)
Semi-annual amplitude (95% CI)	0.002041 (0.001300–0.002855)	0.002665 (0.001197–0.004497)
Annual peak time (weeks, 95% CI)	19.6 (17.0–21.8)	24.7 (0.2–25.9)
Semi-annual periodicity (%, 95% CI)	41.5 (30.0–52.3)	36.4 (25.8–45.1)
Epidemic duration (weeks, 95% CI)	17.0 (11.5–21.0)	21.0 (12.0–29.0)

**Table A5:** Age-specific positive rates in the post-pandemic period and absolute changes compared with the pre-pandemic period, after excluding 2010–2012.

Age Group	Subtype	Post-Pandemic Positive Rates (95% CI)	Absolute Change in Positive Rates (95% CI)	*p*-Value
0–5 m	B/Victoria	11.0 (5.1, 19.8)	9.9 (2.4, 17.5)	<0.001
0–5 m	H1N1pdm09	4.9 (1.3, 12.0)	1.8 (−3.9, 7.5)	0.641
0–5 m	H3N2	6.1 (2.0, 13.7)	4.0 (−2.1, 10.1)	0.1
6–35 m	B/Victoria	2.8 (1.9, 4.0)	1.3 (0.2, 2.3)	0.003
6–35 m	H1N1pdm09	7.6 (6.1, 9.3)	4.3 (2.6, 6.0)	<0.001
6–35 m	H3N2	2.4 (1.6, 3.5)	−1.9 (−3.0, −0.9)	0.003
3–5 y	B/Victoria	4.0 (3.2, 5.0)	0.6 (−0.4, 1.6)	0.226
3–5 y	H1N1pdm09	9.0 (7.8, 10.4)	4.3 (2.9, 5.7)	<0.001
3–5 y	H3N2	5.1 (4.1, 6.1)	−0.7 (−1.8, 0.4)	0.248
6–17 y	B/Victoria	5.2 (4.5, 6.0)	0.8 (−0.1, 1.7)	0.073
6–17 y	H1N1pdm09	9.7 (8.7, 10.7)	4.5 (3.4, 5.6)	<0.001
6–17 y	H3N2	6.2 (5.4, 7.0)	−1.4 (−2.4, −0.4)	0.007

## Appendix B

The median number of tested samples per year is shown as points, with interquartile ranges indicated by lines. Overall, the number of tested samples was similar across the three periods (*p* = 0.159).
